# The Prion Protein N1 and N2 Cleavage Fragments Bind to Phosphatidylserine and Phosphatidic Acid; Relevance to Stress-Protection Responses

**DOI:** 10.1371/journal.pone.0134680

**Published:** 2015-08-07

**Authors:** Cathryn L. Haigh, Carolin Tumpach, Simon C. Drew, Steven J. Collins

**Affiliations:** 1 Department of Medicine (Royal Melbourne Hospital), The University of Melbourne, Melbourne Brain Centre, Melbourne, VIC, AUS, 3010; 2 The Florey Department of Neuroscience and Mental Health, The University of Melbourne, Melbourne Brain Centre, Melbourne, VIC, AUS, 3010; Van Andel Institute, UNITED STATES

## Abstract

Internal cleavage of the cellular prion protein generates two well characterised N-terminal fragments, N1 and N2. These fragments have been shown to bind to anionic phospholipids at low pH. We sought to investigate binding with other lipid moieties and queried how such interactions could be relevant to the cellular functions of these fragments. Both N1 and N2 bound phosphatidylserine (PS), as previously reported, and a further interaction with phosphatidic acid (PA) was also identified. The specificity of this interaction required the N-terminus, especially the proline motif within the basic amino acids at the N-terminus, together with the copper-binding region (unrelated to copper saturation). Previously, the fragments have been shown to be protective against cellular stresses. In the current study, serum deprivation was used to induce changes in the cellular lipid environment, including externalisation of plasma membrane PS and increased cellular levels of PA. When copper-saturated, N2 could reverse these changes, but N1 could not, suggesting that direct binding of N2 to cellular lipids may be part of the mechanism by which this peptide signals its protective response.

## Introduction

The prion protein (PrP^C^) is most widely recognised for its causative association with the transmissible spongiform encephalopathies or prion diseases. During disease PrP^C^ becomes misfolded in a self-templating event that results in its transmissibility. PrP^C^ is a membrane bound (by a glycosylphosphatidylinositol [GPI] anchor), copper-co-ordinating glycoprotein [1]. Aside from GPI-anchor attachment and N-linked glycosylation, PrP^C^ is recognised to undergo further post-translational modifications including at least two endoproteolytic cleavage events termed alpha- and beta-cleavage that result in the formation of N-terminal (N1 and N2) and C-terminal (C1 and C2) fragments respectively [2, 3].

PrP^C^ is localised within cholesterol-rich lipid rafts on the external leaflet of the cell membrane and its interactions with specific lipid species have been linked with both its function and mis-folding [4–9]. The lipid membrane environment is highly important for the control of cellular signal transduction [10] and association of PrP^C^ with lipid raft domains has been shown to be important for its signalling functions [7–9]. The N-terminus of PrP^C^ is known to bind to synthetic lipid membranes [11–13], to target full-length PrP to lipid rafts [14] and to control the movement of PrP^C^ out of lipid raft domains during its copper-induced clathrin mediated internalisation [15, 16]; therefore, the endoproteolytic cleavage events that separate the N and C-termini of PrP^C^ might have a significant impact on its cellular function.

Both the N1 and N2 fragments have been shown to have neuroprotective properties. N1 protects against staurosporine toxicity in cell cultures and ischemia in the rat retina via the p53 pathway [17]. N2 reduces intracellular reactive oxygen species (ROS) production in response to starvation through stimulation of MEK1 signalling in a sequence that depends upon cell surface glycosaminoglycans, intact lipid rafts and copper-dependent endocytosis [18, 19].

Our work has previously shown that synthetic N1 and N2 can bind to lipid membranes containing anionic phospholipids at low pH [12]. Binding is primarily with the lipid head group and does not extend significantly into the acyl tail region, but appears to induce a change in lipid ordering [13]. There is no evidence that these peptides insert into the membrane in such a way as to disrupt membrane integrity, therefore, the peptide-lipid interactions might represent a functional event. This study aimed to determine if N1 and N2 could bind other lipid species and in particular whether such lipid interactions might have a functional influence on the activity of these fragments under the conditions where we have previously seen a neuroprotective action of N2. We identified a previously unknown binding interaction of both N1 and N2 with PA and demonstrated that changes in the cellular lipid environment are associated with cellular stress and the protective action of N2.

## Materials and Methods

### Peptide synthesis

The peptides used and their synthesis have been described previously [13, 18, 20, 21]

### Lipid strip western blotting

Lipid strips were purchased from Life Technologies (Invitrogen; AUS). The protocol provided by the manufacturer was used with the following modifications. Lipid strip incubations were performed in phosphate buffered saline (PBS; Gibco, Invitrogen Life Sciences AUS) at pH 7.0 or in acetate buffer (0.06 g acetic acid, 0.875 g NaCl per 100 ml dH_2_O) at pH 5.0 with 0.5 μg/ml peptide. Equilibration in a sandwich/double blot of new nitrocellulose membrane and blotting paper was assembled in Tris buffer at pH 8 to ensure that if binding to the lipids was pH-dependent the peptide was not washed off the spots during antibody incubations: in this case the peptide would have transferred to the fresh membrane and been detected by blotting. To ensure any lack of detection was not because of epitope masking by the lipid-peptide interaction, western blotting was carried out with two antibodies, SAF32 (1 in 5000; SPI Bio) targeting the octarepeat region residues 79–92 and 8B4 (1 in 1000; Alicon, Switzerland) targeting the N-terminal residues 37–44. Blots and densitometry were carried out as previously [21, 22]. Shorter exposures are shown in S1 Fig to permit comparison of the intensities of the heavy signals in some spots.

### Cell culture

A murine PrP-null neural cell line (CF10) was used throughout. These cells and their culture have been described in detail previously [18, 22, 23].

### Generalised polarisation

Cells were labelled with 5 μM Laurdan (Invitrogen; AUS) in the dark for 30 mins under normal incubator conditions. Fluorescence emission intensity (I) was measured using excitation at 355 nm and emission at 460 and 520 nm. Generalised polarisation (GP) was calculated using the following equation; $GP=\frac{\left(I_{460}-I_{520}\right)}{\left(I_{460}+I_{520}\right)}$. Benzyl alcohol (Sigma-Aldrich, AUS) and filipin III (Sigma-Aldrich), agents known to perturb membranes, were used as a control for increased membrane fluidity.

### NBD-PS labelling

1-oleoyl-2-{12-[(7-nitro-2-1,3-benzoxadiazol-4-yl)amino]dodecanoyl}-*sn*-glycero-3-phosphoserine (ammonium salt; 18:1–12:0 NBD-PS), referred to as NBD-PS throughout, was purchased from Avanti Polar Lipids Inc (USA) and methanol stocks made as per the manufacturer's instructions. Cells were labelled with NBD-PS to a final concentration of 20 μM in ice cold PBS (Gibco, Invitrogen, AUS) with 1 mg/ml bovine serum albumin (BSA; Sigma-Aldrich, AUS) for 2 minutes. Cells were washed once in PBS-BSA before incubation in phenol-red free OptiMEM (Gibco).

### NBD-PS emission spectra

Emission spectrum scans were done following NBD-PS labelling, using 480 nm excitation and 500–600 nm emission in a Cary Eclipse spectrometer (Agilent Technologies, AUS).

### NBD-PS polarisation

Anisotropy was measured following NBD-PS labelling using 470–10 nm excitation and matched 520 nm emission filters in a PolarSTAR Optima plate reader (BMG Labtech AUS).

### Fluorescence microscopy

Cells were imaged as described previously [21].

### Magnetic cell sorting

Cell sorting and counting were performed as previously described [24, 25]. Briefly, cells in suspension were labelled with AnnexinV (which binds with high affinity to externalised phosphatidylserine) magnetic microbeads (Miltenyi Biotech, AUS) and separation in a magnetic field performed as described in the MACS protocol (Miltenyi Biotech). Both labelled (positive) and unlabelled (negative) fractions were transferred into fresh media for counting.

### DCFDA assay

Assays were performed as described previously [22].

### Phospholipase D activity assay

Cells were plated, assayed and harvested in 96-well plates. Lysates were prepared using 20 μl/well 0.1% (v/v) Triton X-100 in assay buffer, followed by three freeze-thaw cycles. Lysate was diluted in a further 30 μl of assay buffer before 1:1 addition of assay reagents as per the Amplex Red Phospholipase D Assay Kit (Invitrogen) product protocol.

### Phosphatidic acid measurement

Cellular PA concentrations were measured using Total Phosphatidic Acid Fluorometric Assay Kit (Cayman Chemical, Sapphire Bioscience, AUS) as per the manufacturer's product protocol with fluorescence measured using a PolarSTAR Optima (BMG). Determination of cellular protein concentration was achieved using the BCA assay as described previously [26].

### Statistical analyses

All graphs represent the mean and SEM. The numerical value for each independent experiment 'n' was the average of the technical replicates. Student's t-test was used for analysis of data with two variables and one-way ANOVA with Tukey's secondary test used for more than two variables. Confidence intervals of 95% were applied for all analyses.

## Results

**Fig 1:**
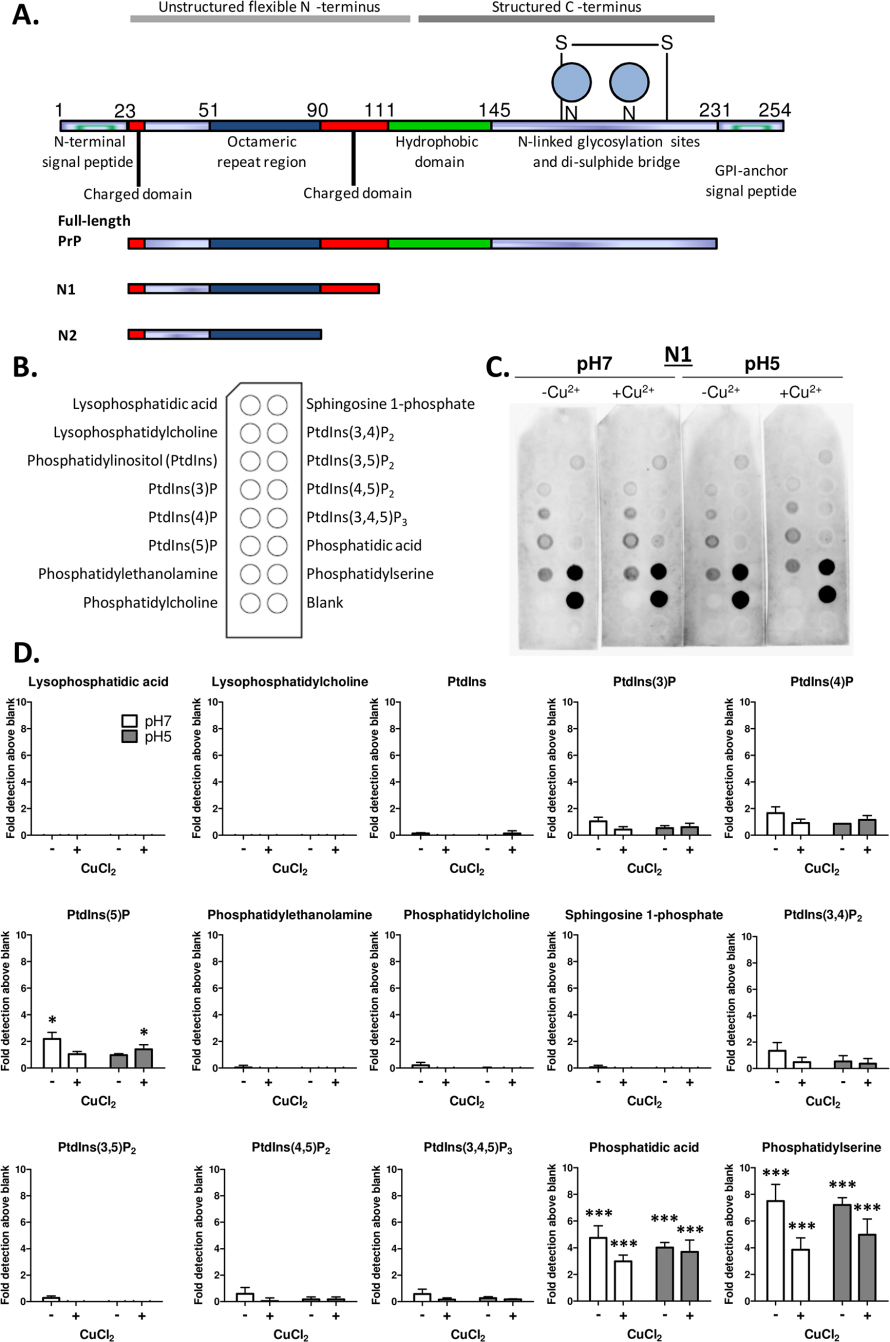
Lipid spot blots identify N1 fragment binding to PS and PA. A. Diagram showing full length PrP and the regions comprising the N1 and N2 cleavage fragments. **B.** Schematic indicating the lipid spot arrangement on the membrane. **C.** PrP23-111 (N1) incubation with the lipid spot blots at pH 7 and pH 5 with and without pre-loading with four molar equivalents CuCl_2_ followed by western blotting with SAF32 antibody (directed against amino acids 51–89). **D.** Densitometric quantification of spot intensity, n = 3, significance over blank control is shown as *p<0.05, ***p<0.001.

**Fig 2:**
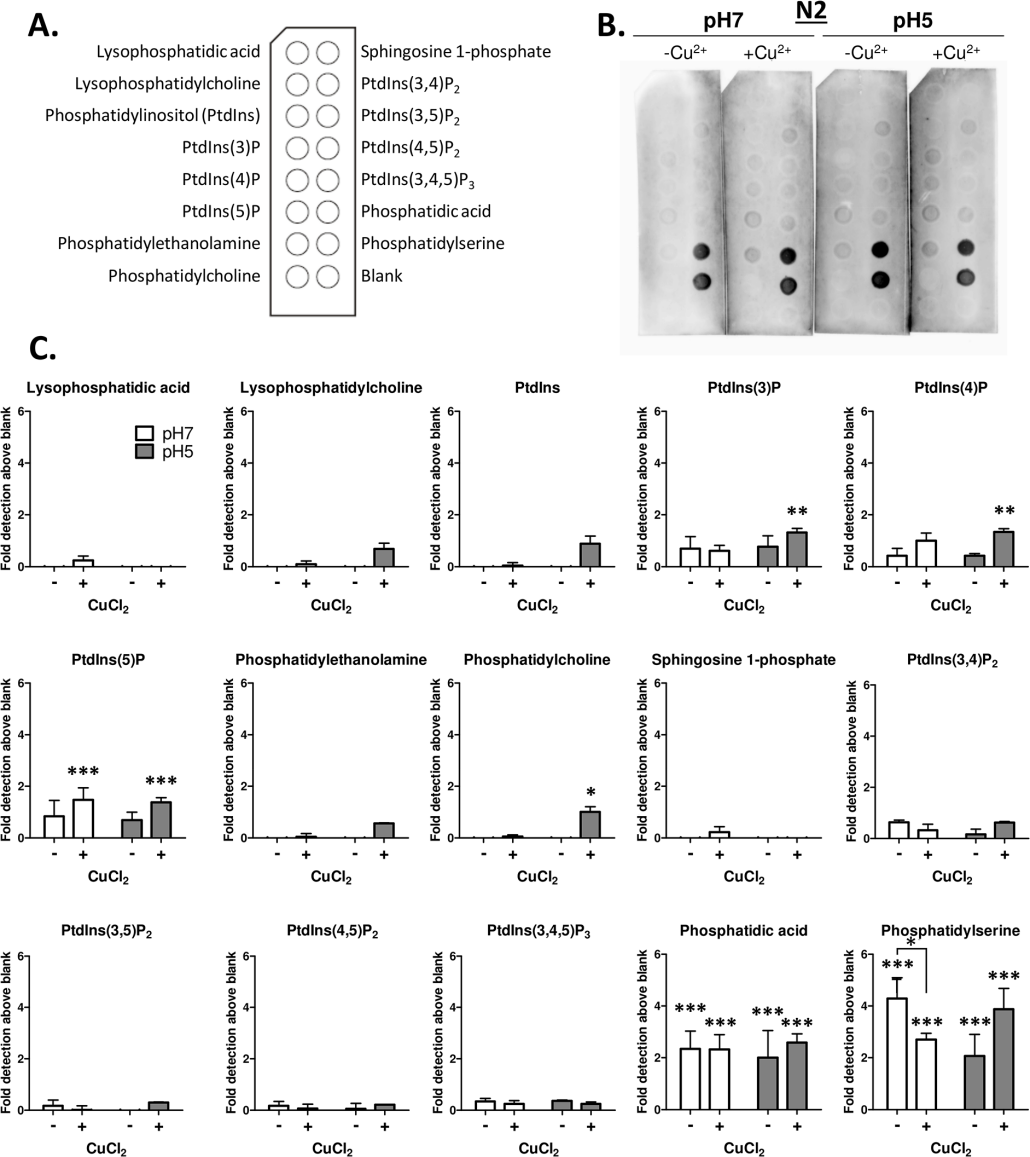
N2 binds PS and PA lipid spots. **A.** Schematic indicating the lipid spot arrangement on the membrane. **B.** PrP23-89 (N2) incubation with the lipid spot blots at pH 7 and pH 5 with and without pre-loading with four molar equivalents CuCl_2_ followed by western blotting with SAF32 antibody (directed against amino acids 51–89). **C.** Densitometric quantification of spot intensity, n = 3, significance over blank control and between conditions is shown as *p<0.05, **p<0.01, ***p<0.001.

Our previous studies using model membranes have shown that N1 and N2 (illustrated in Fig 1A) bind anionic phospholipids at low pH [12, 13]. Such studies are relatively laborious and expensive. Hence to streamline the identification of further interactions with lipids involved in cellular signalling functions we used nitrocellulose spotted with individual lipid species (Fig 1B/2A) and incubated these with synthetically produced N1 (Fig 1) and N2 (Fig 2) at neutral (7) or low (5) pH. The membranes showed that, in accord with results obtained previously, N1 and N2 bound to anionic phosphatidylserine (PS) with limited binding to the neutral phosphatidylcholine (PC). The peptides further showed a strong affinity for phosphatidic acid (PA; Figs 1C&1D, 2B &2C, shorter exposures are shown in S1 Fig). In this assay no pH dependence or loss of peptide binding as membranes were equilibrated to neutral pH was observed (S2 Fig) and binding was identical when detection was made using the octameric repeat region targeting SAF32 antibody and the N-terminally targeted 8B4 (S3 Fig). In our prior studies, N1 bound PS with higher affinity than N2. The densitometry showed that the signal magnitude was higher for the N1 peptide than for the N2, however, the overall binding patterns did not differ (Figs 1D, 2C; plots are shown on the same axis within each peptide analysis for ease of visual comparison of absolute densitometry, for alternate scaling see S4 Fig for N1 and S5 Fig for N2). We additionally assessed the effect of copper binding on these interactions by pre-incubating the N1 and N2 peptides with four molar equivalents CuCl_2_ before incubation with the membranes. These results indicated that copper and pH together may weakly, but not significantly, enhance binding to some lipid species but significantly decrease N2 binding to PS at pH 7.

**Fig 3:**
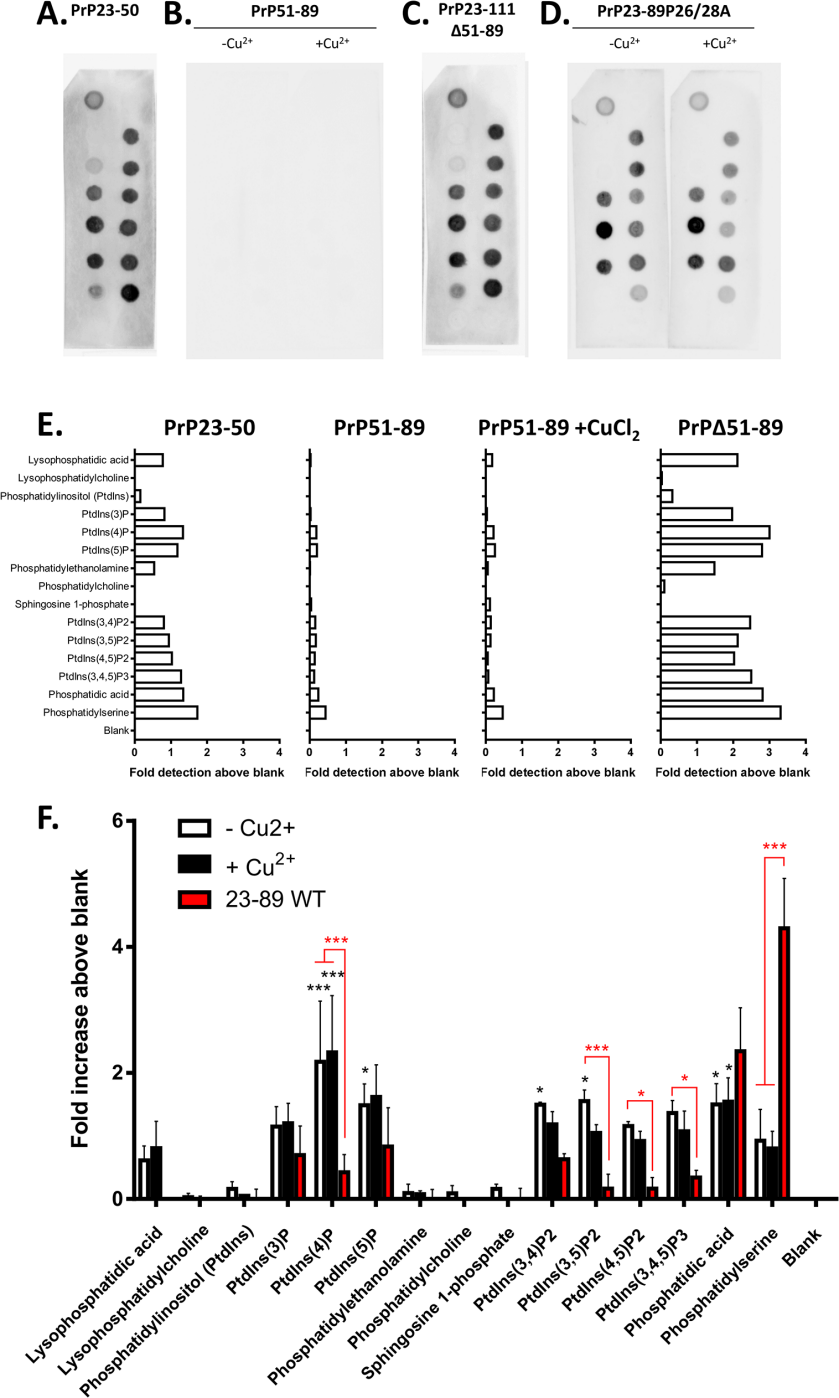
Lipid binding specificity is determined by regions of N1 and N2. Lipid spot blots (incubated at pH 7) of peptides corresponding to regions of N1 and N2, including residues 23–50 (**A**), residues 51–89 comprising the copper-binding region and therefore tested with and without copper saturation (**B**) and an N1 fragment lacking the residues of the copper-binding region, Δ51–89 (**C**). For 23–50 and N1Δ51–89, copper saturation was not tested as neither fragment contains the octarepeat copper-binding domain and blotting used the N-terminally targeted 8B4 antibody as SAF32 targets residues 79–92. **D.** Lipid spot blots of a mutant N2 P26/28A fragment with and without copper saturation at pH 7 detected with SAF32. **E.** Densitometric quantification of spot intensity for the domains of N1 and N2, n = 1. **F.** Densitometric quantification of spot intensity of the P26/28A mutation of N2 with and without copper saturation. Apo N2 intensities are shown for comparison of differences between the mutated and wild-type (WT) sequence, n = 3. Significance over blank control is shown in black and significant differences in detection from the wild type sequence of N2 are shown in red, *p<0.05, ***p<0.001.

To examine the importance of each region of the N-terminal fragments for PS and PA binding we incubated the membrane with peptides containing residues 23–50 (Fig 3A) and 51–89 octameric repeat region (Fig 3B), plus a peptide representing N1 minus the 51–89 copper-binding domain (Fig 3C) and a peptide representing N2 with the proline residues at amino acids 26 and 28 mutated to alanine (PrP23-89P26/28A; Fig 3D). The octameric repeat region alone was unable to bind to any lipid regardless of whether it was loaded with Cu^2+^ (Fig 3B/3E; a longer exposure of these strips alongside the P26/28A peptide for comparison is shown in S6 Fig and alternatively scaled axes are shown in S7 Fig). Absence of the octameric repeat region from N2, leaving only residues 23–50 (Fig 3A/3E) or deletion of the octameric repeat region from N1 (Fig 3C/3E) Fig led to a greater number of interactions with different lipids. Mutation of the two proline residues within the polybasic N-terminus (Fig 3D/3F) also resulted in a promiscuity of lipid binding with significant changes from the wild type N2 sequence seen for phosphatidylinositol (4)P, (3,5)P2, (4,5)P2, (3,4,5)P3 and PS. From these observations, we may conclude that the specificity of lipid binding by N1 and N2 requires the full complement of N-terminal and octarepeat residues.

**Fig 4:**
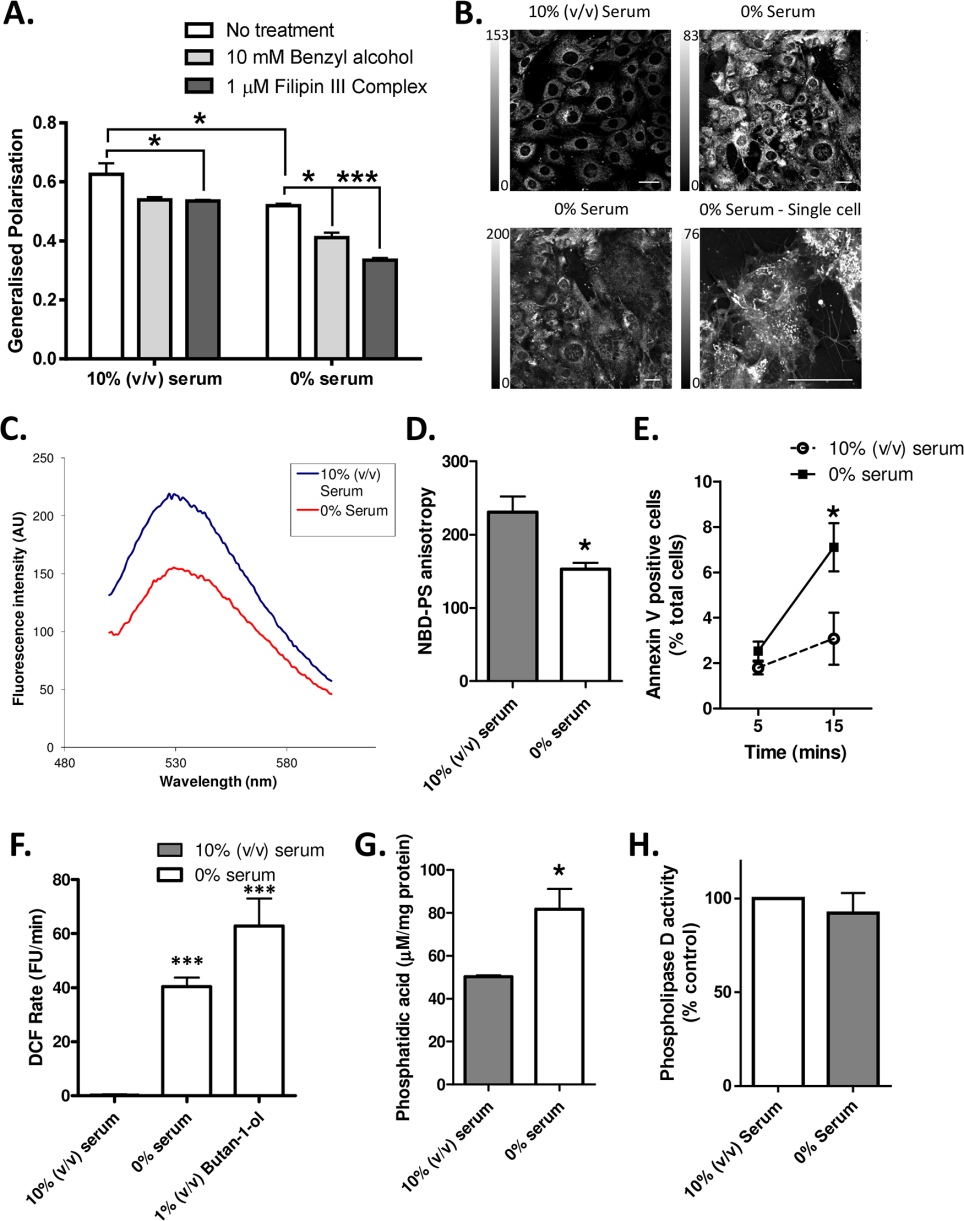
Serum deprivation causes changes to PS and PA in CF10 cells. **A.** Laurdan GP changes following serum deprivation for 30 minutes as compared with benzyl alcohol (BA) and filipin III controls. n = 3. **B.** Live cell imaging of NBD-PS labelled CF10 cells. Image intensity is thresholds have been selected to view detail in the staining pattern and do not represent a comparison of fluorescence intensity. Scale bars = 20 μm. **C.** Fluorescence emission spectra of NBD-PS labelled cells following transfer into serum-free medium, scans were taken immediately after media replacement. **D.** Anisotropy of NBD-PS in CF10 cells with and without serum present. n = 3. **E.** Counts from magnetic separation of cells that have lost membrane asymmetry allowing them to bind PS at 5 and 15 minutes post serum withdrawal. n = 4. **F.** ROS production detected by DCF fluorescence when cells are serum-starved and with exposure to butan-1-ol to inhibit PLD activity. n = 4. **G.** Measurement of cellular phosphatidic acid concentration 30 minutes after commencing serum deprivation. n = 3. **H.** Measurement of phospholipase-D activity following 15 minutes serum starvation. n = 3. For all panels, *p<0.05, ***p<0.001.

Our previous studies have found that copper-bound N2 normalises cellular responses to the stress of nutrient starvation [18, 21]. To assess whether alterations in the lipid membrane environment may be involved in the cellular response to starvation, changes in the fluorescence profile of an environmentally sensitive probe, Laurdan, were assessed using generalised polarisation (GP; the principle of this assay is described in [27, 28]). The GP value upon serum deprivation showed that decreased phospholipid order within the cell membrane occurred in response to nutrient withdrawal (Fig 4A). As both peptides were shown to bind PS and PA, changes to each of these lipids were also specifically assessed. Incorporation of PS containing a NBD fluorescent label within the fatty acid chain (NBD-PS) into cultured CF10 cells and microscopic examination of the NBD-PS cells following serum withdrawal revealed a broader spatial distribution of NBD-PS fluorescence (Fig 4B). Furthermore, using this same probe it was found that upon serum withdrawal the fluorescence intensity of the probe and its anisotropy are decreased (Fig 4C & 4D). In similarity with the environmentally sensitive Laurdan probe, NBD is quenched in more aqueous environments [29], therefore, these results indicate that during serum deprivation the cell membrane is becoming less ordered and more fluid.

It is also known that an early indicator of apoptosis is loss of membrane asymmetry at the cell surface, which results in abnormal external exposure of PS and might contribute to some of the changes detected by NBD-PS. Using the affinity of Annexin-V for PS, binding of Annexin-V coated magnetic beads was employed to separate cells with exposed PS from those cells maintaining membrane asymmetry. These separations revealed a significant increase in the number of cells exposing PS in the nutrient deprived population after 15 minutes of starvation (Fig 4E).

PA is generated from catalysis of PC by phospholipase D (PLD) at the plasma membrane and acts as a second messenger in various signalling pathways [30, 31]. Therefore to look for the role of PA in signalling the cellular response to serum starvation, butan-1-ol was used to inhibit the action of this enzyme. Our previous studies have shown that serum deprivation induces a large and rapid production of reactive oxygen species (ROS) within cells [18, 21]. We confirmed this production and assessed the effect of butan-1-ol on ROS production, finding that the butan-1-ol stimulated an even greater increase in intra-cellular ROS (Fig 4F). Butan-1-ol might elicit non-specific cellular actions and therefore, to confirm whether cellular PA production was changed following serum deprivation, we examined the cellular concentration of PA thirty minutes after serum withdrawal. At this time PA concentrations in the serum deprived cells were almost double those of the cells incubated under normal conditions (Fig 4G). Measurement of PLD activity showed no significant change in enzyme activity (Fig 4H), indicating that the changed levels of PA measured might be a result of decreased degradation of PA.

**Fig 5:**
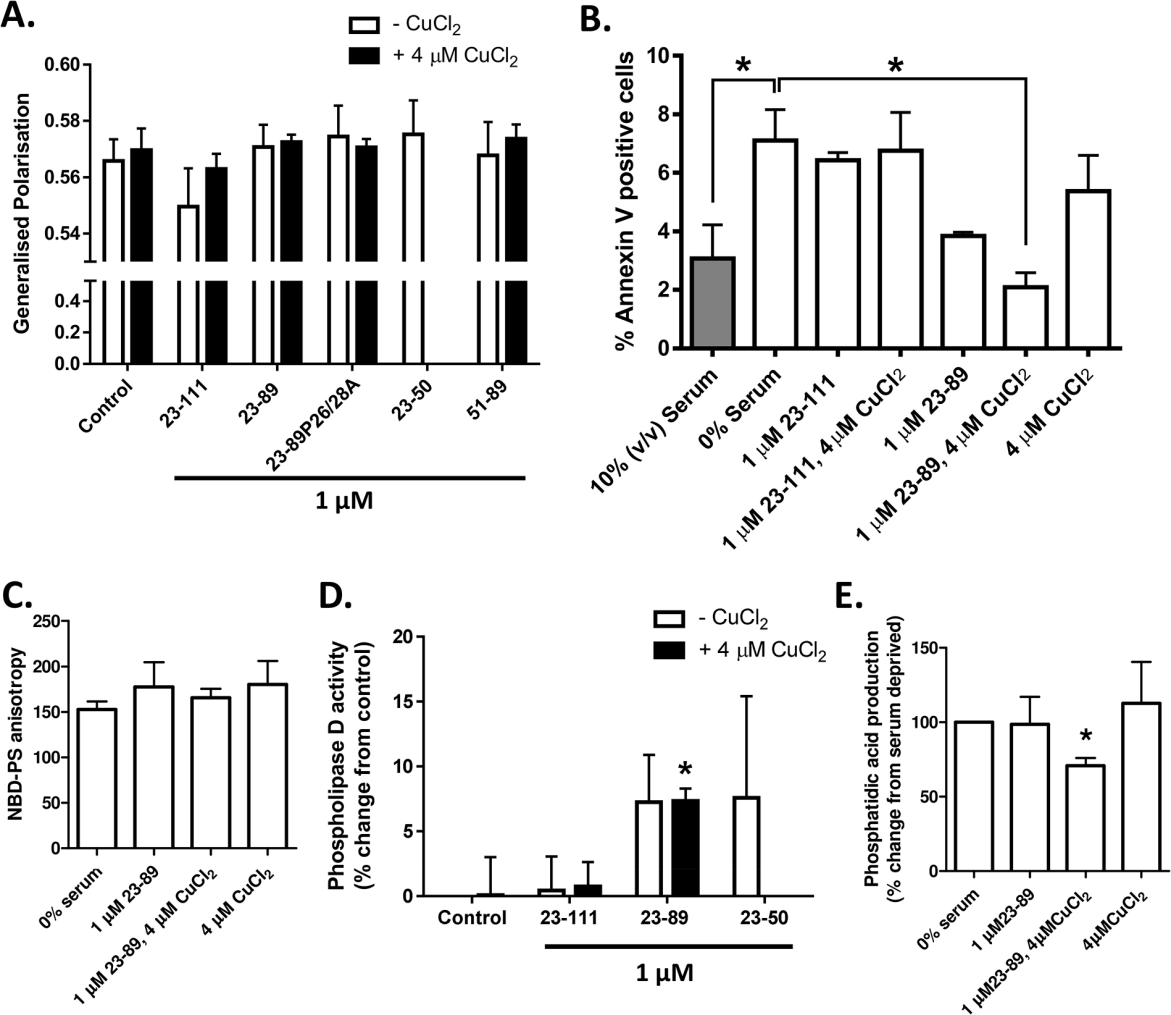
N2, but not N1, reverses PS externalisation and PA increase in the absence of further membrane changes. **A.** Laurdan GP of serum starved cells alone and when treated N2 (23–89), N1 (23–111), N2 with the two prolines within the N-terminal polybasic region mutated to alanines (23-89P26/28A, the octarepeat region (51–89), copper-saturated (four molar equivalents) N1, N2, 23-89P26/28A and 51–89, 23–50 and equivalent copper without peptide, measured under the same conditions used in Fig 4. n = 3. **B.** Annexin V magnetic separation of N1 and N2 with and without copper saturation. Filled bar indicates 10% (v/v) serum and hollow bars show conditions with 0% serum. n = 3. **C.** NBD-PS anisotropy of N2 with and without copper saturation. n = 3. **D.** Phospholipase-D activity within N1 and N2 (+/- copper), and 23–50 (no copper) serum starved cells. n = 3. **E.** Relative PA concentrations within N2 treated (+/- copper) serum starved cells. n = 3. Fig *p<0.05.

To determine if the protective actions of copper-loaded N2 [18] or the previously observed neuroprotective actions of N1 [17] might involve or affect the membrane lipid environment, the Laurdan GP assay was repeated comparing the effects of N1 and N2 (+/- copper saturation) with the GP values of serum deprived cells alone. Neither peptide significantly influenced laurdan GP (Fig 5A), showing they are unable to influence overall membrane fluidity. To more specifically look at PS and PA we assessed the influence of serum deprivation in the presence or absence of N1 and N2 (with and without pre-loading with copper). Annexin-V separations of cells following serum deprivation showed that copper-saturated N2, but not N1, reversed the loss of asymmetry induced by serum deprivation (Fig 5B). Despite the influence of copper saturated N2 on PS externalisation, but in agreement with the results seen for laurdan generalised polarisation, NBD-PS anisotropy was unchanged (Fig 5C). Such findings indicate that whilst the externalisation of the PS is restored in response to copper-loaded N2, the changes in fluidity caused by serum deprivation are not reversed.

In contrast with the unchanged PLD activity in response to serum deprivation, copper loaded N2 significantly increased the activity of PLD under these conditions (Fig 5D). Apo-N2 and 23–50 produced highly variable (not significant) fluctuations in PLD activity and N1 showed no change from baseline, regardless of copper saturation. To assess the consequences of the increased PLD activity stimulated by copper-saturated N2, cellular concentrations of PA were assessed. Despite the increased enzymatic activity of PLD, copper-saturated N2 counteracted increases in PA induced by serum deprivation (Fig 5E) suggesting an increased turnover of PA, which requires enzymatic compensation. An alternative interpretation of the data is that N2 binding to PA impairs its enzymatic digestion into the end products detected in the analysis, resulting in an apparently reduced PA level due to artefactual under-detection.

## Discussion

Whilst the binding of the N1 and N2 fragments to PS is well established in model membrane systems [12, 13], to the best of our knowledge an interaction between any region of PrP^C^ and PA has never been reported. Both the 51–89 amino acid copper-binding region and the proline motif within the basic amino acids at the far N-terminus were required for the specificity of the interaction with PS and PA. This observation could be highly significant in view of the fact that the previously reported protective effect of the N2 fragment also required both of these domains [18]. Weak binding was seen for several of the other lipid species, however this is unlikely to be of relevance in a cellular context where higher affinity binding partners would be able to out-compete and dominate.

Our previous studies into N1 and N2 lipid binding found that interactions with PS-containing bilayers could only occur at low pH [12, 13], which was not the case for the lipid strip assays in the current study. A potential explanation for the discrepancy between the data lies in the methodology. The lipid strips are prepared from pure spots of a single lipid species, immobilised on a nitrocellulose membrane [32, 33]. Consequently they are intended as a screening tool and are not designed to give specific information on interactions that require free movement of lipids such as that provided by the complex biophysical techniques used in our previous studies. However, they did correctly detect the previously indentified interaction with PS and inability to bind PC; therefore, it is highly likely that the interaction with PA will occur under the right conditions. The specifics of these conditions remain to be investigated.

Assessment of the serum deprived cells showed that the lipid environment was consistently perturbed and that such changes occurred rapidly. As there is no evidence of membrane lysis during starvation (despite externalisation of PS these cells do not die [21]), such results are indicative of changed lipid order. Therefore, under conditions of nutrient withdrawal, PS externalisation may not be entirely a pro-apoptotic event but a cellular compensation to the stress of starvation and it is in this context that N1 and N2 binding could be significant. Under normal cellular conditions PS is localised to the inner leaflet of the cell membrane and PrP^C^ is on the outer leaflet, where the N2 cleavage is proposed to occur [34, 35]. Further, the N1 and N2 fragments are consistently found to be secreted from cells into the surrounding milieu [17, 36–39]. Therefore, the localisation of each molecule is usually so disparate that they would be unlikely to interact. However, during cellular stress, an opportunity exists for N1 and/or N2 to bind and modulate PS function.

Within the current study copper-saturated N2 reduced the percentage of cells with externalised PS. Whilst the data presented herein cannot rule out that N2 directly binds to and blocks the annexin-V binding site, resulting in an apparently reduced detection, our data suggests that this is unlikely as the same response was not seen for N1, which has a stronger affinity for PS membranes than N2 [12, 13] and the bound N2 would have to survive trypsin digest. The difference in cellular response to N1 and N2 suggests that they mediate their protective effects by engaging different pathways or that the protective actions of each peptide are induced by different stimuli. Such differences likely stem from the additional charged domain of N1, which may increase its affinity for a non-lipid target in preference and/or addition to functionally binding the identified lipid species.

The interaction of N1 and N2 with PA is of special interest since PA is a known signalling intermediate that feeds into the ras-MEK-ERK pathway [30]. The changes in PLD activity and PA levels following treatment with the copper-saturated N2 suggest that it could be modulating this pathway through cellular PA changes. N2 stress-protective signalling has recently been shown to be transduced through MEK1 and requires copper-induced internalisation of the peptide [21]. PA production is also associated with endocytosis through its stimulation of membrane curvature, fission and fusion [40] and it has further been shown to regulate clathrin induced endocytosis [41]. Clathrin induced internalisation of full-length PrP^C^ is mediated by its N-terminus [15, 16], with this region being present in both the N1 and N2 fragments. Therefore, membrane association of N2 and PA could represent a genuine functional engagement activating a larger signalling complex or cascade.

Within the lipid spot binding assay copper appeared to have very subtle effects on the intensity of peptide binding. However N2 copper saturation was required for significant changes to be observed in both the annexin-V positive counts and in changes to PA production and levels. This suggests that, whilst not altering N2 affinity for lipids, acts as an essential co-factor, mediating the interactions between N2, lipids and other receptors involved in the transduction of cellular stress responses.

## Conclusions

The findings of the current study showed that the N-terminal endoproteolytic cleavage products of PrP, N1 and N2, demonstrated marked binding to PS and PA and that this interaction required the full complement of N-terminal and octarepeat residues. Additionally, copper-saturated N2 demonstrated some capacity to normalise starvation-induced cellular changes to both lipids. Therefore, PS and PA feasibly represent direct targets or pathway intermediates by which N2 transduces its neuroprotective functions.

## Supporting Information

S1 Fig Lipid strip staining as revealed by shorter exposure times. **A.** Schematic showing the spot arrangement on the membrane. Eight second exposure of **B)** N1 membranes, **C)** N1 membranes and **D)** N1/N2 domain fragment membranes (30 second exposures are shown in the main text).
(PDF)

S2 Fig Double blots of N2 spot blots following equilibration to neutral pH. Membranes were equilibrated in Tris buffer (pH 8) in a blotting paper—lipid spot membrane-fresh membrane—blotting paper sandwich before western blotting for N2 on both the original and new membranes with saf32 antibody. Almost no detectible transfer onto the new membrane (shown) was evident indicating the peptide remained bound to the lipid spot membrane as pH was changed.
(PDF)

S3 Fig N1 and N2 lipid spot blots probed with 8B4 antibody. Blots were carried out exactly as for Saf32 blotting with 8B4 used as the detection antibody. The similarity in staining pattern using SAF32 (Fig 1B and 1D) and 8B4 (below) shows that detection of an interaction is not being missed by epitope masking.
(PDF)

S4 Fig Rescaled N1 lipid spot binding plots. Plots shown in Fig 1 of the main text have been re-scaled on an axis appropriate to their signal intensity. No bars indicates no detectable signal on any repeat, n = 3.
(PDF)

S5 Fig Rescaled N2 lipid spot binding plots. Plots shown in Fig 2 of the main text have been re-scaled on an axis appropriate to their signal intensity. No bars indicates no detectable signal on any repeat, n = 3.
(PDF)

S6 Fig Extended exposures of lipid spot blots incubated with the PrP 51–89 peptide. All blots shown in the main text are 30 second exposures. For incubations with a peptide comprising residues 51–89 (the octarepeat copper-binding region), membranes were blank after 30 seconds so a further 5 minute exposure was done alongside the strongly labelled mutant 23-89P26/28A peptide. After the five minute exposure a small degree of labelling was evident for the 51–89 peptide was comparatively very weak against the signal seen for the 23–89 P26/28A peptide.
(PDF)

S7 Fig Rescaled plots of N1/N2 domain fragments. Plots shown in Fig 3 of the main text have been re-scaled on an axis appropriate to their signal. No bars indicates no detectable signal, n = 1.
(PDF)
