# Supplementary material for: The Prion Protein N1 and N2 Cleavage Fragments Bind to Phosphatidylserine and Phosphatidic Acid; Relevance to Stress-Protection Responses
Source: PLoS One. 2015 Aug 7;10(8):e0134680. doi: 10.1371/journal.pone.0134680 (PMC4529310; doi:10.1371/journal.pone.0134680)

Supplementary Figure S1. *Lipid strip staining as revealed by shorter exposure times.* **A.** Schematic showing the spot arrangement on the membrane. Eight second exposure of **B)** N1 membranes, **C)** N1 membranes and **D)** N1/N2 domain fragment membranes (30 second exposures are shown in the main text).

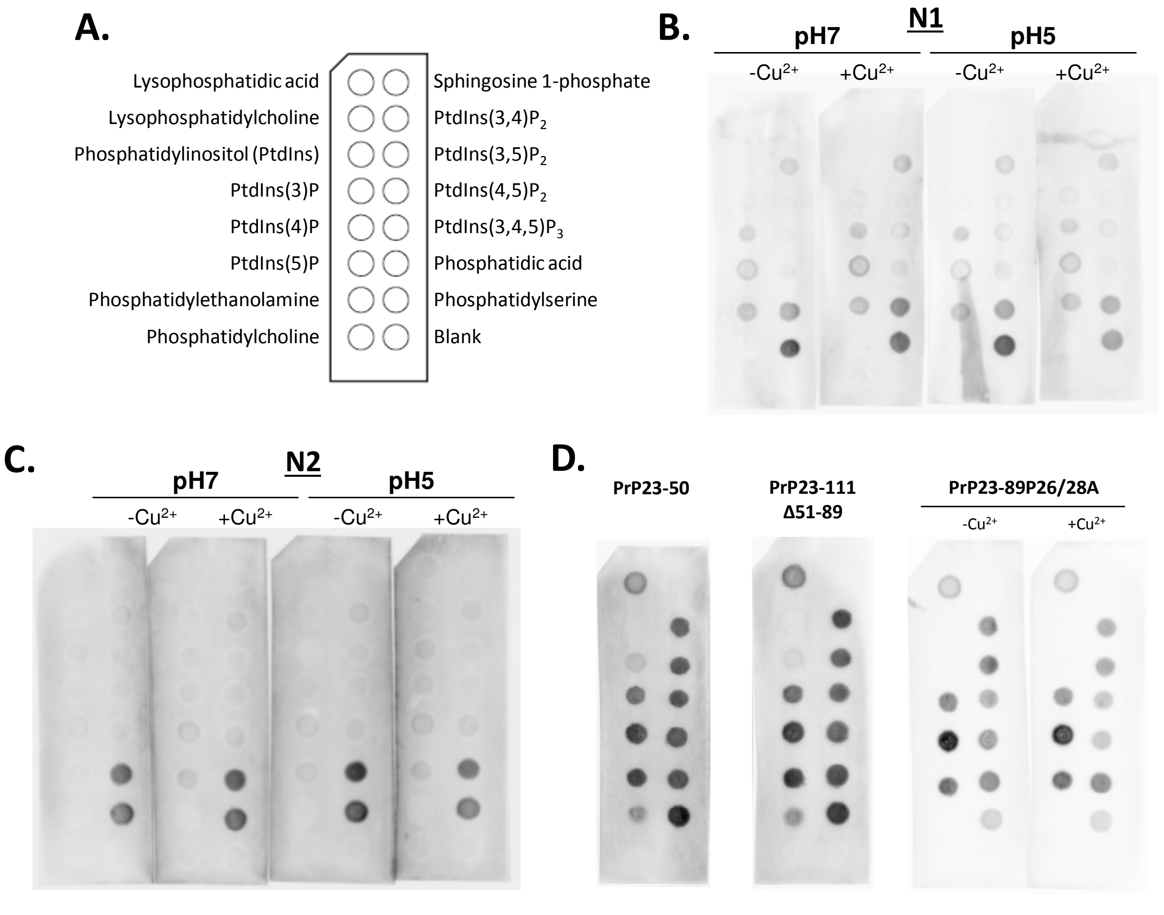

Supplement: S1 Fig — A. Schematic showing the spot arrangement on the membrane. Eight second exposure of B) N1 membranes, C) N1 membranes and D) N1/N2 domain fragment membranes (30 second exposures are shown in the main text). (PDF) [file pone.0134680.s001.pdf]
