# Supplementary material for: The Prion Protein N1 and N2 Cleavage Fragments Bind to Phosphatidylserine and Phosphatidic Acid; Relevance to Stress-Protection Responses
Source: PLoS One. 2015 Aug 7;10(8):e0134680. doi: 10.1371/journal.pone.0134680 (PMC4529310; doi:10.1371/journal.pone.0134680)

Supplementary Figure S2. *Double blots of N2 spot blots following equilibration to neutral pH.* Membranes were equilibrated in Tris buffer (pH 8) in a blotting paper - lipid spot membrane - fresh membrane - blotting paper sandwich before western blotting for N2 on both the original and new membranes with saf32 antibody. Almost no detectible transfer onto the new membrane (shown) was evident indicating the peptide remained bound to the lipid spot membrane as pH was changed.

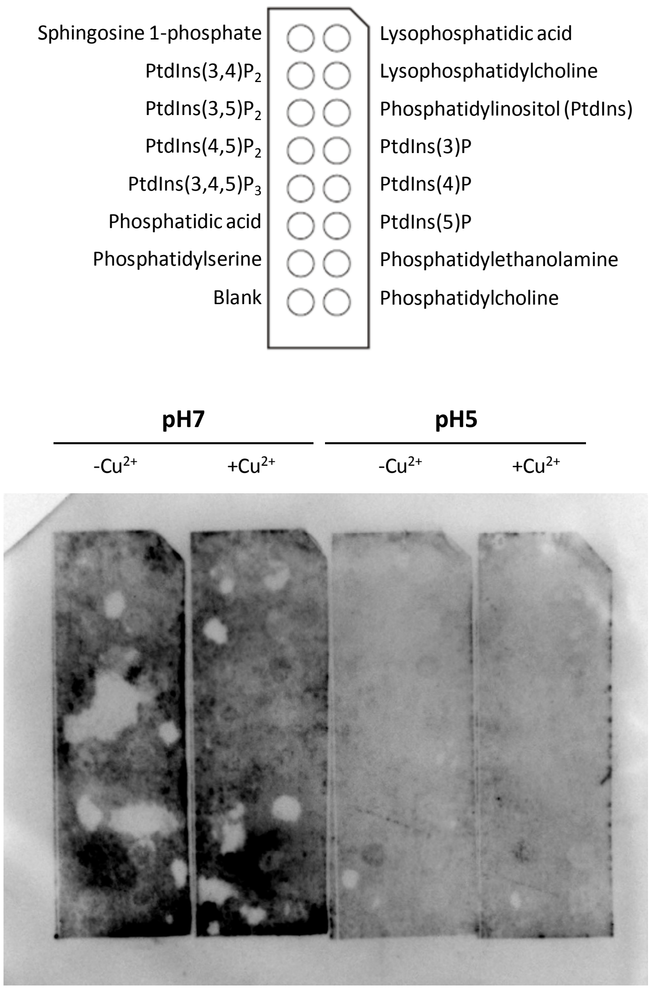

Supplement: S2 Fig — Membranes were equilibrated in Tris buffer (pH 8) in a blotting paper—lipid spot membrane-fresh membrane—blotting paper sandwich before western blotting for N2 on both the original and new membranes with saf32 antibody. Almost no detectible transfer onto the new membrane (shown) was evident indicating the peptide remained bound to the lipid spot membrane as pH was changed. (PDF) [file pone.0134680.s002.pdf]
