# Supplementary material for: The Prion Protein N1 and N2 Cleavage Fragments Bind to Phosphatidylserine and Phosphatidic Acid; Relevance to Stress-Protection Responses
Source: PLoS One. 2015 Aug 7;10(8):e0134680. doi: 10.1371/journal.pone.0134680 (PMC4529310; doi:10.1371/journal.pone.0134680)

Supplementary Figure S3. *N1 and N2 lipid spot blots probed with 8B4 antibody.* Blots were carried out exactly as for Saf32 blotting with 8B4 used as the detection antibody. The similarity in staining pattern using SAF32 (Fig 1B, D) and 8B4 (below) shows that detection of an interaction is not being missed by epitope masking.

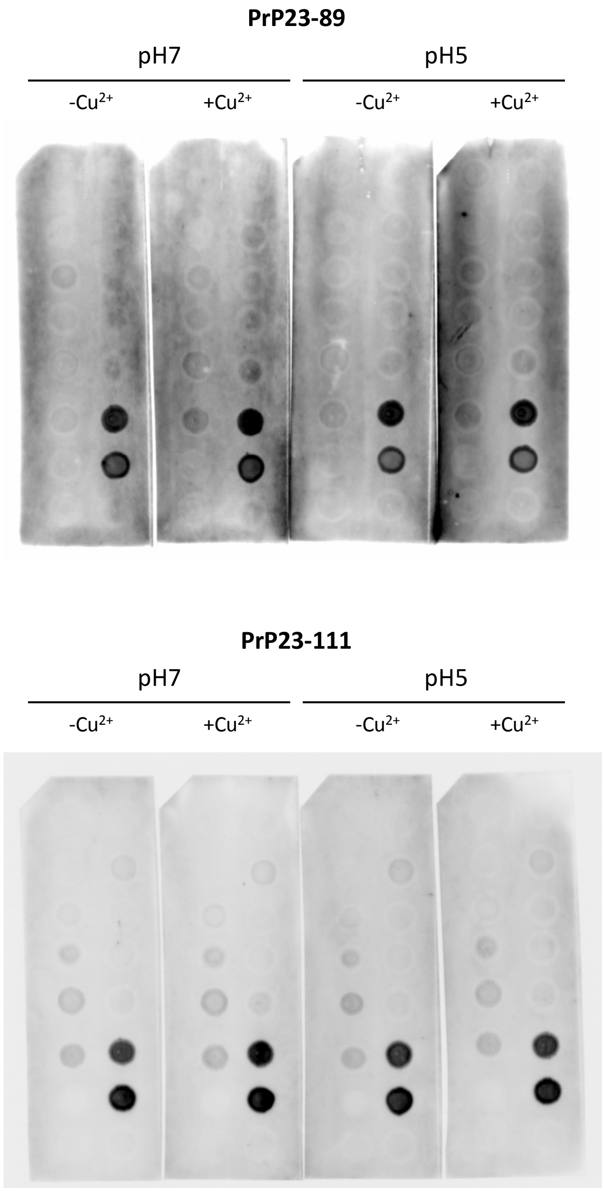

Supplement: S3 Fig — Blots were carried out exactly as for Saf32 blotting with 8B4 used as the detection antibody. The similarity in staining pattern using SAF32 (Fig 1B and 1D) and 8B4 (below) shows that detection of an interaction is not being missed by epitope masking. (PDF) [file pone.0134680.s003.pdf]
