# Supplementary material for: The Prion Protein N1 and N2 Cleavage Fragments Bind to Phosphatidylserine and Phosphatidic Acid; Relevance to Stress-Protection Responses
Source: PLoS One. 2015 Aug 7;10(8):e0134680. doi: 10.1371/journal.pone.0134680 (PMC4529310; doi:10.1371/journal.pone.0134680)

Supplementary Figure S4. *Rescaled N1 lipid spot binding plots.* Plots shown in figure 1 of the main text have been re-scaled on an axis appropriate to their signal intensity. No bars indicates no detectable signal on any repeat, n = 3.

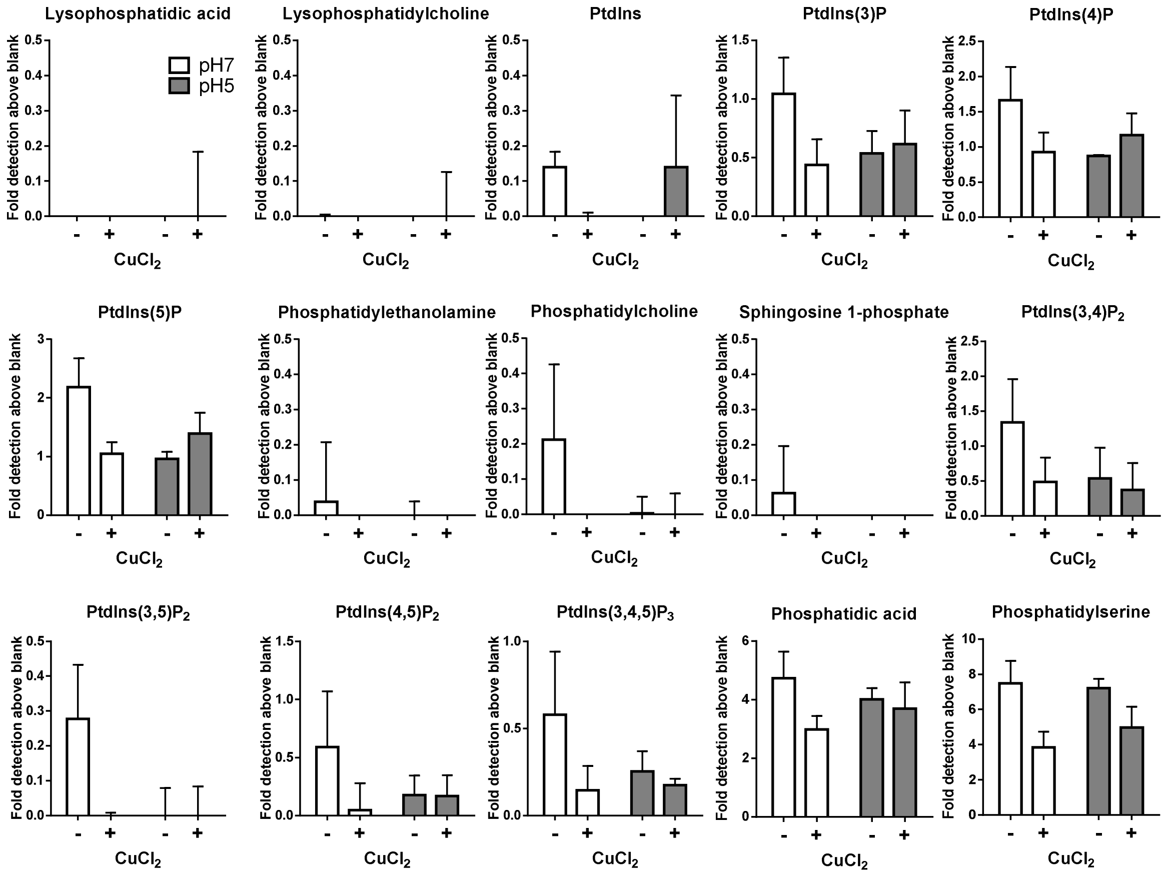

Supplement: S4 Fig — Plots shown in Fig 1 of the main text have been re-scaled on an axis appropriate to their signal intensity. No bars indicates no detectable signal on any repeat, n = 3. (PDF) [file pone.0134680.s004.pdf]
