# Supplementary material for: The Prion Protein N1 and N2 Cleavage Fragments Bind to Phosphatidylserine and Phosphatidic Acid; Relevance to Stress-Protection Responses
Source: PLoS One. 2015 Aug 7;10(8):e0134680. doi: 10.1371/journal.pone.0134680 (PMC4529310; doi:10.1371/journal.pone.0134680)

Supplementary Figure S5. *Rescaled N2 lipid spot binding plots.* Plots shown in figure 2 of the main text have been re-scaled on an axis appropriate to their signal intensity. No bars indicates no detectable signal on any repeat, n = 3.

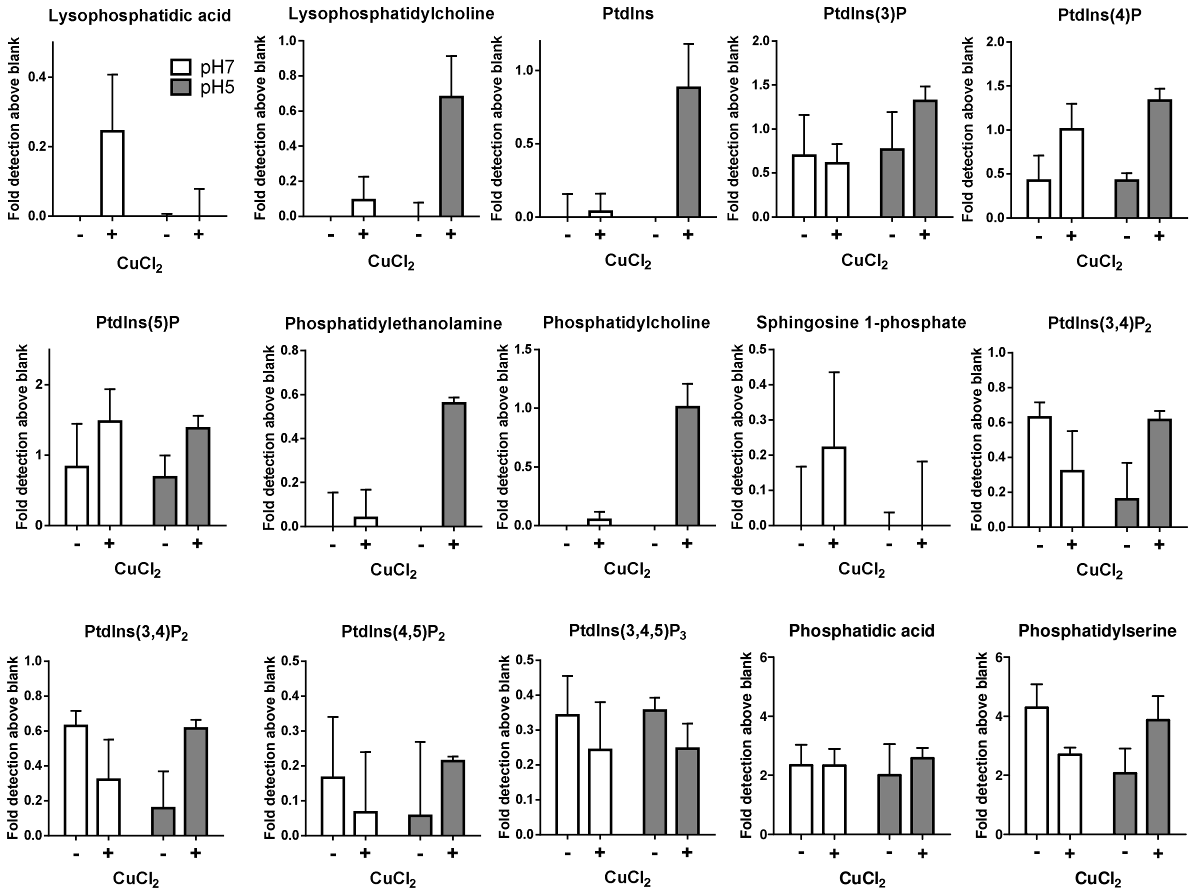

Supplement: S5 Fig — Plots shown in Fig 2 of the main text have been re-scaled on an axis appropriate to their signal intensity. No bars indicates no detectable signal on any repeat, n = 3. (PDF) [file pone.0134680.s005.pdf]
