# Supplementary material for: The Prion Protein N1 and N2 Cleavage Fragments Bind to Phosphatidylserine and Phosphatidic Acid; Relevance to Stress-Protection Responses
Source: PLoS One. 2015 Aug 7;10(8):e0134680. doi: 10.1371/journal.pone.0134680 (PMC4529310; doi:10.1371/journal.pone.0134680)

Supplementary Figure S6. *Extended exposures of lipid spot blots incubated with the PrP 51-89 peptide.* All blots shown in the main text are 30 second exposures. For incubations with a peptide comprising residues 51-89 (the octarepeat copper-binding region), membranes were blank after 30 seconds so a further 5 minute exposure was done alongside the strongly labelled mutant 23-89P26/28A peptide. After the five minute exposure a small degree of labelling was evident for the 51-89 peptide was comparatively very weak against the signal seen for the 23-89 P26/28A peptide.

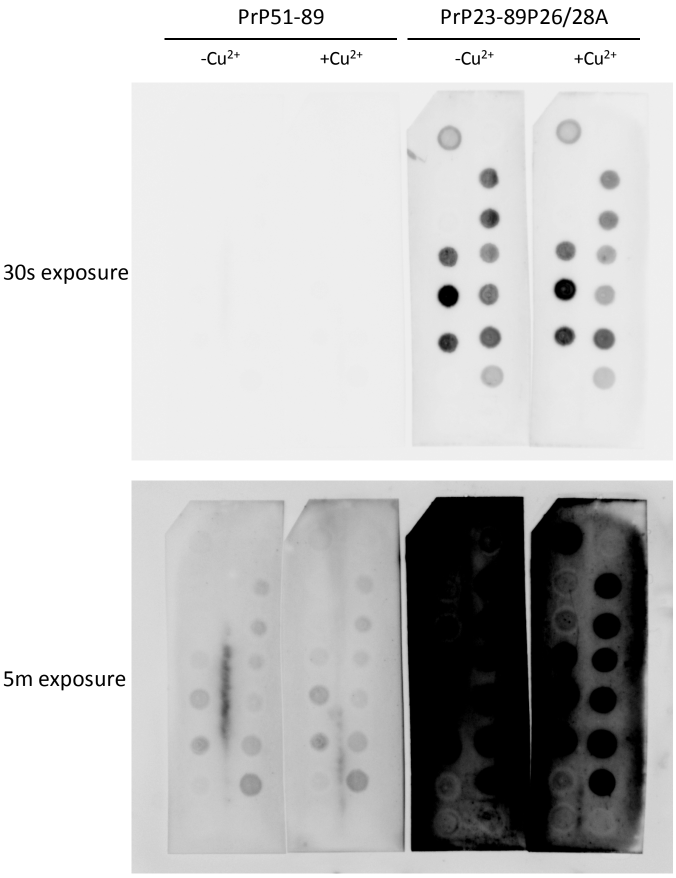

Supplement: S6 Fig — All blots shown in the main text are 30 second exposures. For incubations with a peptide comprising residues 51–89 (the octarepeat copper-binding region), membranes were blank after 30 seconds so a further 5 minute exposure was done alongside the strongly labelled mutant 23-89P26/28A peptide. After the five minute exposure a small degree of labelling was evident for the 51–89 peptide was comparatively very weak against the signal seen for the 23–89 P26/28A peptide. (PDF) [file pone.0134680.s006.pdf]
